# Supplementary figures and images for: Dissecting disease tolerance in Plasmodium vivax malaria using the systemic degree of inflammatory perturbation
Source: PLoS Negl Trop Dis. 2021 Nov 2;15(11):e0009886. doi: 10.1371/journal.pntd.0009886 (PMC8589215; doi:10.1371/journal.pntd.0009886)

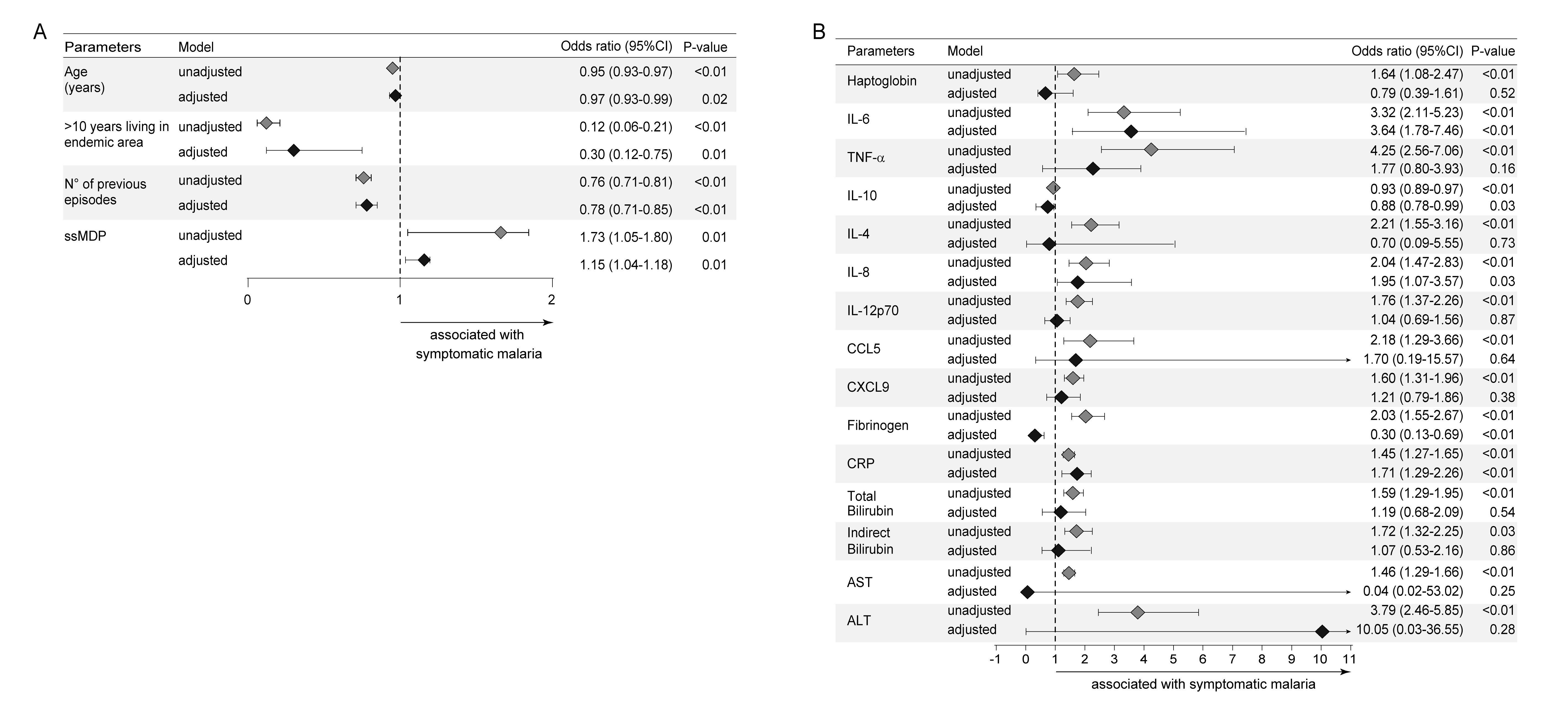

Supplement: S1 Fig — Adjusted multinomial logistic regression analysis was performed with symptomatic malaria as the primary outcome. The model was composed with variables that were statistically significant (p < 0.05) in univariate comparisons (see univariate comparisons in Table 1). (TIF) [file pntd.0009886.s002.tif]

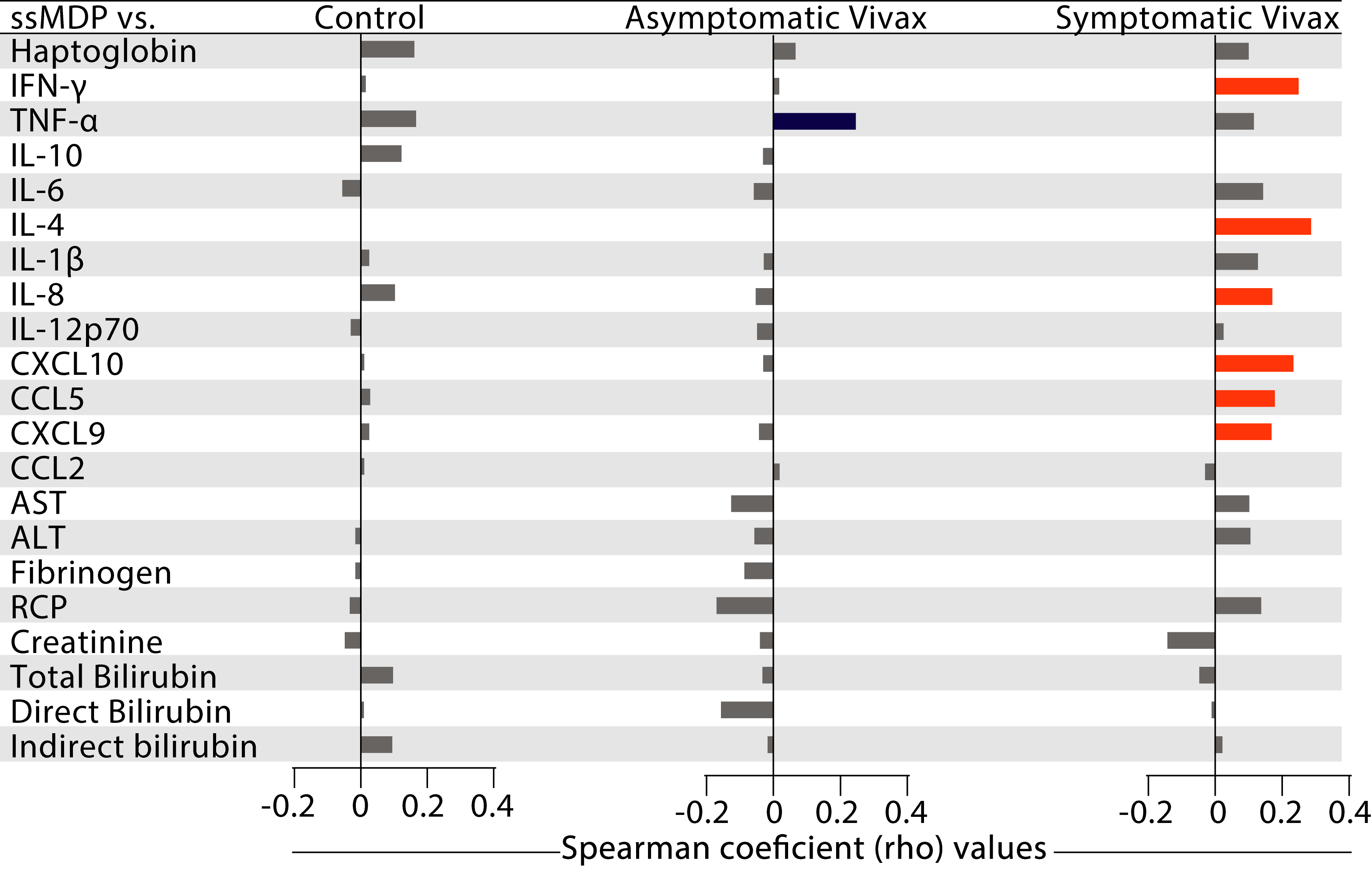

Supplement: S2 Fig — A Spearman correlation analysis was employed to identify the perturbation of each individual marker that contribute to changes in the systemic inflammatory imbalance, assessed by ssMDP score values, in each group as indicated. Colored bars infer the correlation with P-value<0.05 after adjustment for multiple comparisons. Abbreviations (alphabetic order): ALT: alanine aminotransferase; AST: aspartate aminotransferase; CCL: C-C motif chemokine ligand; CXCL: C-X-C motif chemokine ligand; CRP: C-reactive protein; IFN: interferon; IL: interleukin; TNF: tumor necrosis factor. (TIF) [file pntd.0009886.s003.tif]

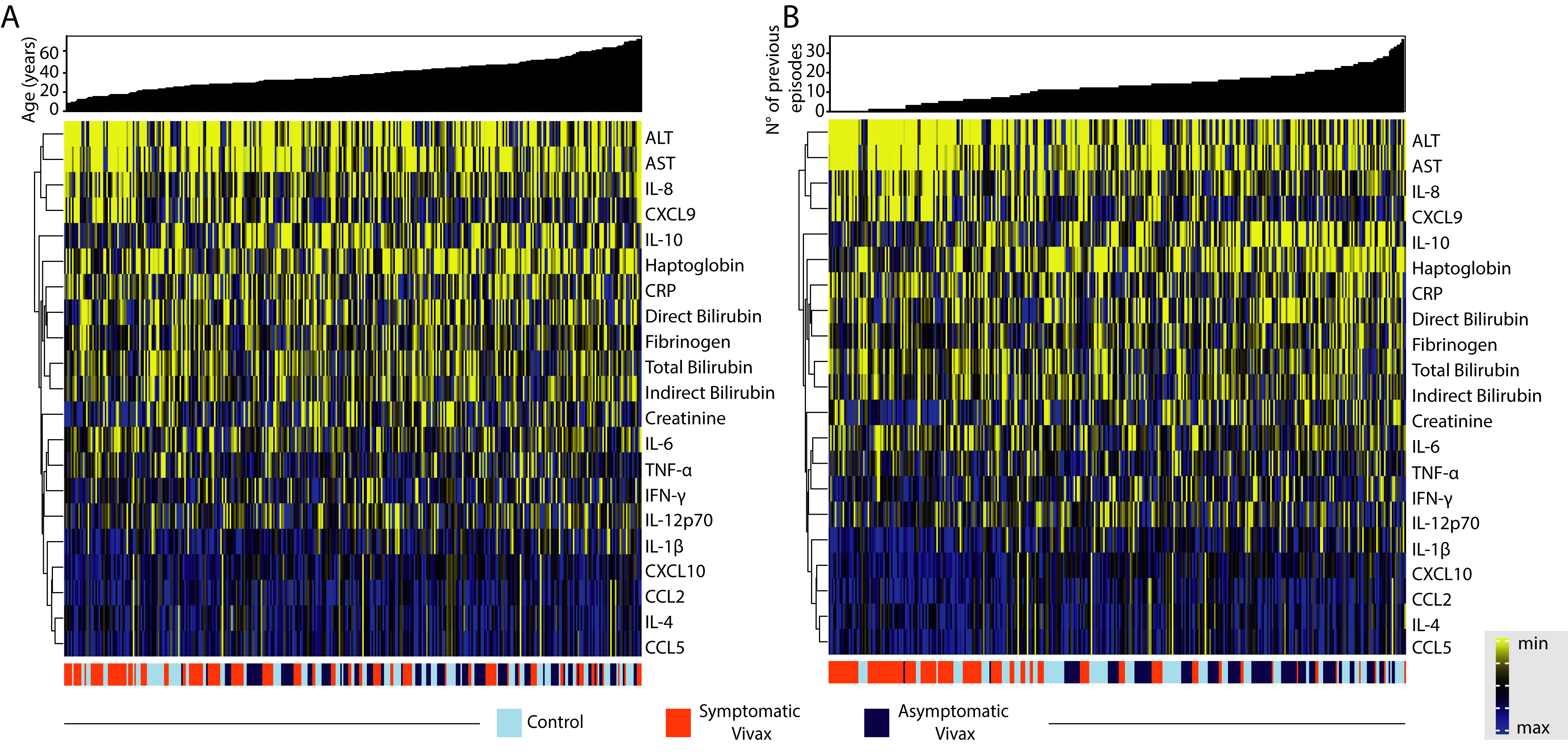

Supplement: S3 Fig — Hierarchical cluster analysis of Log-10 transformed and z-score normalized using Ward’s method with 100X bootstrap was employed to depict the overall perturbation of inflammatory and biochemical markers in study population. The participants were grouped based on age (A) and number of previous Malaria episodes (B). (TIF) [file pntd.0009886.s004.tif]
